# Combined application of BNLF2b antibody with VCA-IgA, Rta-IgG, and Zta-IgA in nasopharyngeal carcinoma screening in the Guangxi region

**DOI:** 10.1371/journal.pone.0332606

**Published:** 2025-09-22

**Authors:** Ruilan Lin, Ru Qin, Yunlong Zhang, Yao Guan, Boheng Wu, Shangyang Li, Shenhong Qu, Yulin Yuan

**Affiliations:** 1 Guilin Medical University, Guilin, Guangxi, China; 2 Department of Laboratory Medicine, The People’s Hospital of Guangxi Zhuang Autonomous Region, Nanning, Guangxi Zhuang Autonomous Region, China; 3 Department of Otolaryngology & Head and Neck, People’s Hospital of Guangxi Zhuang Autonomous Region, Nanning, Guangxi, China; Fondazione Don Carlo Gnocchi, ITALY

## Abstract

**Background:**

This study aims to assess the diagnostic value of the Epstein-Barr virus (EBV) BNLF2b antibody(P85-Ab), alone or in combination with VCA-IgA, Rta-IgG, and Zta-IgA antibodies, in the context of nasopharyngeal carcinoma (NPC).

**Methods:**

The study included 100 NPC patients and 100 healthy controls. Chemiluminescent microparticle immunoassay was utilized to measure P85-Ab levels in the serum samples of both NPC patients and healthy controls. Additionally, the ELISA method was employed to detect serum levels of VCA-IgA, Rta-IgG, and Zta-IgA antibodies. The study analyzed the roles of serum P85-Ab in conjunction with VCA-IgA, Rta-IgG, and Zta-IgA antibodies in the diagnosis of NPC.

**Results:**

Serum levels of P85-Ab, VCA-IgA, Rta-IgG, and Zta-IgA antibodies in NPC patients were significantly higher than those in the normal control group (P < 0.05). The area under the receiver operating characteristic curve (AUC) for individual diagnosis of NPC using serum P85-Ab, VCA-IgA, Rta-IgG, and Zta-IgA antibodies were 0.964, 0.916, 0.838, and 0.840, respectively. The AUC for the combined detection of the four markers in diagnosing NPC was 0.996, indicating the optimal diagnostic value of the combined detection.

**Conclusion:**

The combined detection of P85-Ab with VCA-IgA, Rta-IgG, and Zta-IgA antibodies demonstrates high diagnostic value for nasopharyngeal carcinoma. Serum P85-Ab may serve as a potential marker for the diagnosis of NPC.

## Introduction

Nasopharyngeal carcinoma, a type of cancer that originates from the mucosal epithelium of the nasopharynx, is characterized by subtle clinical manifestations [1,2]. This cancer exhibits varying incidence rates across different demographics, with a notably high occurrence in regions such as southern China and Southeast Asia [2–6]. The World Health Organization has classified nasopharyngeal carcinoma into three subtypes based on keratinization and tumor differentiation [7]. Despite research efforts to uncover the underlying causes of this cancer, the exact etiology remains incompletely elucidated [8]. Current knowledge suggests associations with genetic predisposition, Epstein-Barr virus infection, and environmental exposures [8].

Radiotherapy, a cornerstone in treating nasopharyngeal carcinoma, significantly impacts patient outcomes [9]. Recent advancements in radiotherapy technology have led to a notable increase in the 5-year survival rate, reaching approximately 90% for early-stage patients but dropping below 50% for those in advanced stages [10]. Consequently, early and accurate detection plays a critical role in nasopharyngeal carcinoma.

The primary methods for diagnosing nasopharyngeal carcinoma involve biopsies and various imaging techniques, given the anatomical intricacies of the nasopharynx. Early detection in this region poses considerable challenges due to its unique structure. The occurrence of distant metastasis in nasopharyngeal carcinoma frequently occurs in later stages, lacking distinct initial symptoms or indicators, thereby detrimentally affecting clinical outcomes. Hence, the exploration of biomarkers for diagnosing nasopharyngeal carcinoma holds critical clinical relevance.

The field of nasopharyngeal carcinomsa screening has witnessed significant advancements in recent years. These developments have encompassed the integration of a diverse array of biomarkers, the implementation of multi-stage screening processes, and the discovery of novel biomarkers through research efforts [11–16]. While these strides represent important progress, the current landscape is still characterized by challenges such as limited sensitivity, the complexity of screening procedures, and the high costs associated with existing methods. As a result, urgent attention is required to delve into uncharted territories in search of fresh biomarkers and innovative strategies that can offer heightened sensitivity and robust positive predictive value for the effective screening of nasopharyngeal carcinoma. Given the relatively low incidence of nasopharyngeal carcinoma cases, achieving a notably high level of specificity is crucial to ensure a meaningful positive predictive value in screening protocols.

P85-Ab is a promising antibody biomarker for nasopharyngeal carcinoma. Recent investigations have uncovered that the serological detection of nasopharyngeal carcinoma has benefited from the high specificity (99.6%) and sensitivity (94.4%) exhibited by P85-Ab, making them a valuable tool for supplementary diagnostic purposes [17]. To enhance the accuracy of detection, this research assesses the performance of P85-Ab tested separately and in combination with VCA-IgA, Rta-IgG, and Zta-IgA.

The primary objective of this study is to investigate the diagnostic potential utility of P85-Ab, both independently and when combined with VCA-IgA, Rta-IgG, and Zta-IgA antibodies, in the context of nasopharyngeal carcinoma.

## Materials and methods

### Ethics

Approval for this research was granted by the Ethics Committee at the People’s Hospital of Guangxi Zhuang Autonomous Region under Ethics No. 2021-KY-139–01, ensuring compliance with the ethical guidelines set forth by the committee. Prior to participation, all individuals (or their legal guardians) provided informed consent.

### Samples

A cohort of 100 individuals diagnosed with nasopharyngeal carcinoma (NPC) at the People’s Hospital of Guangxi Zhuang Autonomous Region between December 30, 2021, and November 30, 2022, were chosen for the study. Concurrently, 100 healthy subjects without tumor-related illnesses, matched for the same timeframe at the identical hospital, were enlisted as the control group.

Inclusion criteria encompassed: 1) initial diagnosis of nasopharyngeal carcinoma, devoid of prior chemotherapy or alternative anti-cancer therapies; 2) possession of comprehensive imaging and clinical records; 3) expression of willingness to provide informed consent; 4) absence of anomalies in the healthy controls. Patients meeting any of the subsequent exclusion criteria were omitted from the research: 1) individuals who had undergone radiation therapy or chemotherapy; 2) individuals with severe liver or kidney impairment, pulmonary disorders, critical cardiovascular conditions, diabetes, or additional malignancies; 3) individuals with acute infections or hematologic disorders; 4) pregnant or lactating females.

Data pertaining to demographic and clinical attributes of the patients were amassed, encompassing age, sex, World Health Organization (WHO) pathological grading, American Joint Committee on Cancer (AJCC) (8th edition) [18]clinical staging, TNM staging, treatment status.

### Detection (testing for P85-Ab, VCA-IgA, Rta-IgG, Zta-IgA)

Venous blood samples of approximately 3 mL were collected from all participants and centrifuged at 3000 rpm for 10 minutes to isolate serum. Serum samples from 100 nasopharyngeal carcinoma patients and 100 healthy individuals were tested for P85-Ab antibodies using a fully automated chemiluminescent immunoassay analyzer (Wan200 + model), manufactured by Xiamen Youmaike Medical Instrument Co., Ltd. The EBV P85-Ab detection kit was provided by Xiamen Wantai Kairui Biological Technology Co., Ltd., and the assay was conducted in strict accordance with the manufacturer’s instructions. The serum volume used for detection was automatically managed by the system.

VCA-IgA, Rta-IgG, and Zta-IgA antibody levels were measured using enzyme-linked immunosorbent assay (ELISA) kits. Specifically, the VCA-IgA kit was supplied by Beijing Xinxing Sihuan Biotechnology Co., Ltd., the Rta-IgG kit by Beijing Tongxin Bioengineering Co., Ltd., and the Zta-IgA kit by Zhongshan Bioengineering Co., Ltd. For each test, 10 µL of serum was used for VCA-IgA and Rta-IgG, and 5 µL of serum for Zta-IgA. All three ELISA assays were performed on a fully automated ELISA analyzer (URANUS AE 275, Shenzhen iKang Biotechnology Co., Ltd.), following the respective manufacturers’ instructions.

## Data analysis

Statistical analyses were conducted utilizing SPSS software version 16.0. Graphs were generated with SPSS 16.0 and GraphPad Prism 9.5. Measurement data, determined to be non-normally distributed, were presented as medians and analyzed using the rank-sum test. Count data were represented as rates and analyzed through the chi-square test. For comparisons of sensitivity between individual antibodies or antibody combinations, unpaired chi-square tests were performed. Specificity, Youden Index, PPV, and NPV are presented descriptively. A significance threshold of P < 0.05 was used.

Combinations of biomarkers were evaluated using binary logistic regression models. For each combination, the presence of specific biomarkers was treated as independent variables, and disease status as the dependent variable. Predicted probabilities were computed based on the logistic regression outputs and were subsequently used to construct Receiver Operating Characteristic (ROC) curves. These ROC curves were used to assess the diagnostic performance of each combination of biomarkers, including metrics such as the Area Under the Curve (AUC), sensitivity, and specificity. This approach allowed for a comprehensive evaluation of the combined diagnostic potential of multiple biomarkers in the detection of nasopharyngeal carcinoma.

## Results

### 1. Characteristics of participants

In the cohort of 100 participants, 75 were male and 25 were female, averaging 48.24 years old (range: 14 to 74 years). According to WHO classification and AJCC staging, 47% were at stage III, and 48% at stage IV (Table 1).

**Table 1:** Characteristics of 100 Participants in Nasopharyngeal Carcinoma (NPC).

Characteristics	No.of patients	Positive for Rta	Positive for VCA	Positive for Zta	Positive for P85-Ab
Total	100	73	90	62	92
AJCC stage					
I	3	3	1	2	3
II	2	0	2	2	2
III	47	36	44	26	43
IV	48	34	43	32	44
Tumor stage					
T1	16	13	13	10	16
T2	28	18	27	17	26
T3	30	26	29	17	26
T4	26	16	21	18	24
Nodal stage					
N0	7	5	4	4	5
N1	6	2	4	5	5
N2	62	46	57	36	59
N3	25	20	25	17	23
Metastatic stage					
M0	85	62	77	52	77
M1	15	11	13	10	15

The control group of 100 healthy individuals included 53 males and 47 females, averaging 45.94 ± 10.32 years old (range: 25 to 64 years). Comparing the age and gender distribution between nasopharyngeal carcinoma patients and controls showed no significant differences (P > 0.05), indicating comparability.

### 2. Diagnostic value of P85-Ab, VCA-IgA, Rta-IgG, and Zta-IgA antibody detection for nasopharyngeal carcinoma

**Fig 1:**
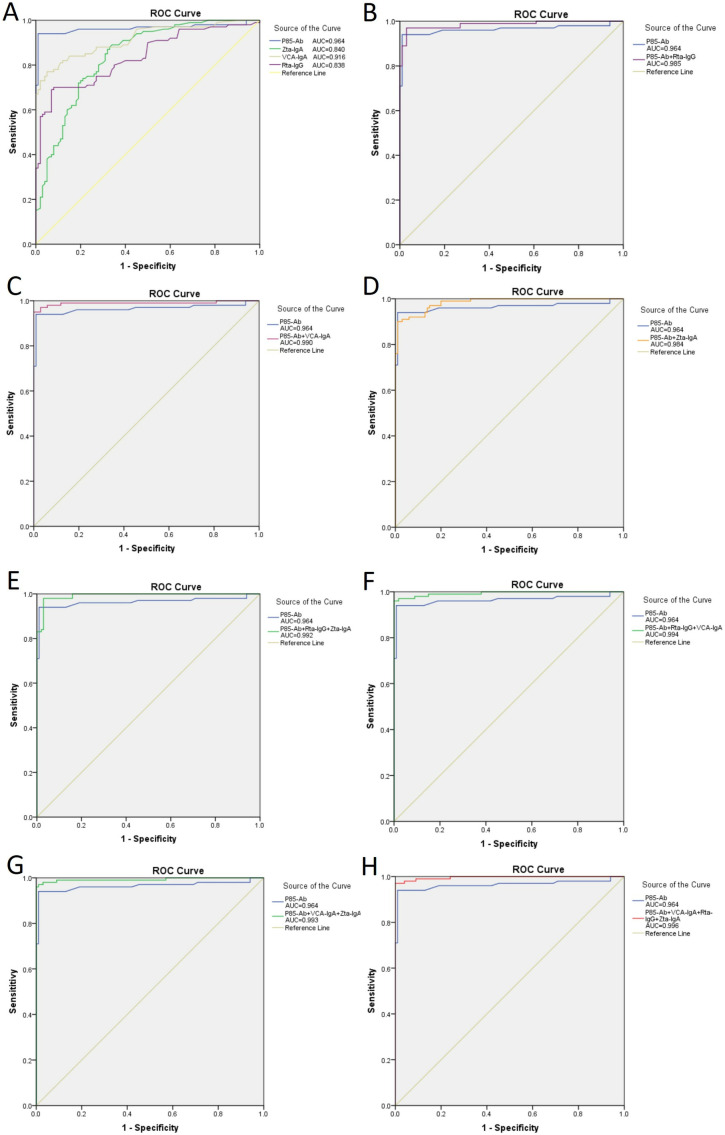
Diagnosis efficacy. Diagnosis efficacy of P85-Ab, Rta-IgG, VCA-IgA and Zta-IgA in the diagnosis of nasopharyngeal carcinoma (NPC).

The results of the four antibody tests are presented in Table 2. The sensitivity, specificity, positive predictive value, negative predictive value, and Youden index for P85-Ab antibody detection were 93.00%, 99.00%, 98.94%, 93.40%, and 0.92, respectively. For Rta-IgG antibody detection, these values were 59.00%, 96.00%, 93.65%, 70.07%, and 0.55, respectively. The sensitivity, specificity, positive predictive value, negative predictive value, and Youden index for Zta-IgA antibody detection were 62.00%, 88.00%, 83.78%, 69.84%, and 0.50, respectively. For VCA-IgA antibody detection, these values were 75.00%, 95.00%, 93.75%, 79.17%, and 0.70, respectively. The diagnostic performance of the four antibody tests was evaluated using ROC curve analysis. Moreover, when directly compared with other single biomarkers, P85-Ab demonstrated significantly superior diagnostic efficiency (all P < 0.05) (Table 2). Furthermore, ROC curve analysis indicated that P85-Ab antibody detection exhibited high diagnostic efficiency for nasopharyngeal carcinoma (Fig 1).

**Table 2:** Diagnostic values of P85-Ab, Rta-IgG, VCA-IgA and Zta-IgA for NPC.

Antibody Test	Sensitivity(%) (95%CI)	Specificity(%) (95%CI)	Youden Index (95%CI)	PPV (%) (95%CI)	NPV (%) (95%CI)	P
P85-Ab	93.00 (89.47-96.53)	99.00 (97.62-100.00)	0.92 (0.88-0.96)	98.94 (98.31-99.57)	93.40 (88.54-98.26)	—
Rta-IgG	59.00 (52.17-65.83)	96.00 (93.29-98.71)	0.55 (0.48-0.62)	93.65 (92.14-95.16)	70.07 (63.74-76.40)	<0.05
Zta-IgA	62.00 (55.27-68.73)	88.00 (83.51-92.49)	0.50 (0.42-0.58)	83.78 (78.31-89.25)	69.84 (63.20-76.48)	<0.05
VCA-IgA	75.00 (69.80-80.20)	95.00 (92.80-97.20)	0.70 (0.63-0.77)	93.75 (89.74-97.76)	79.17 (73.64-84.70)	<0.05

**Note:** P values indicate comparison with the reference group (P85-Ab). P < 0.05 was considered statistically significant. “—” indicates the reference group. P values refer to sensitivity comparisons using unpaired chi-square tests.

### 3. Diagnostic value of combined antibody detection for nasopharyngeal carcinoma

Incorporating combined antibody detection results in Table 3, the ROC curve analysis underscores the improved sensitivity of the tests while upholding satisfactory specificity levels. The data are visually represented in Fig 1, enabling direct observation and interpretation of the findings.(Fig 1)

**Table 3:** Diagnostic values of Rta-IgG, Zta-IgA, P85-Ab and VCA-IgA for NPC.

Combination	Sensitivity(%) (95%CI)	Specificity(%) (95%CI)	Youden Index (95%CI)	PPV (%) (95%CI)	NPV(%) (95%CI)	P
P85-Ab	93.00 (89.47-96.53)	99.00 (97.62-100.00)	0.92 (0.88-0.96)	98.94 (98.31-99.57)	93.40 (88.54-98.26)	—
P85-Ab + Rta-IgG	97.00 (94.60-99.40)	97.00 (94.60-99.40)	0.94 (0.91-0.97)	97.00 (94.60-99.40)	97.00 (94.60-99.40)	>0.05
P85-Ab + Zta-IgA	90.00 (85.80-94.20)	99.00 (97.60-100.00)	0.89 (0.85-0.93)	98.90 (94.30-100.00)	90.83 (86.80-94.80)	>0.05
P85-Ab + VCA-IgA	97.00 (94.60-99.40)	99.00 (97.60-100.00)	0.96 (0.93-0.99)	98.98 (94.52-100.00)	97.06 (93.70-100.00)	>0.05
P85-Ab + Rta-IgG + Zta-IgA	97.00 (94.60-99.40)	97.00 (94.60-99.40)	0.95 (0.92-0.98)	97.00 (94.60-99.40)	98.00 (96.03-99.97)	>0.05
P85-Ab + VCA-IgA + Rta-IgG	97.00 (94.60-99.40)	98.00 (96.03-99.97)	0.95 (0.92-0.98)	97.98 (96.05-99.91)	97.03 (94.60-99.46)	>0.05
P85-Ab + VCA-IgA + Zta-IgA	97.00 (94.60-99.40)	99.00 (97.60-100.00)	0.96 (0.93-0.99)	98.98 (94.52-100.00)	97.06 (93.70-100.00)	>0.05
P85-Ab + Rta-IgG + Zta-IgA + VCA-IgA	98.00 (96.03-99.97)	96.00 (93.33-98.69)	0.94 (0.91-0.97)	96.08 (93.40-98.76)	97.96 (95.15-100.00)	0.088

**Note:** P values indicate comparison with the reference group (P85-Ab alone) using the Chi-square test. P < 0.05 was considered statistically significant. P values between 0.05 and 0.1 suggest a positive trend: although not formally significant, the combination may provide some improvement. Notably, the P value for the four combined indicators was 0.088, close to marginal significance “—” indicates the reference group. P values refer to sensitivity comparisons using unpaired chi-square tests.

## Discussion

In China, nasopharyngeal carcinoma, a malignancy originating in the epithelial cells of the nasopharynx, exhibits high incidence and mortality rates [1,2]. The Epstein-Barr virus (EBV), a herpesvirus infecting human lymphocytes, plays a significant role in the initiation and progression of this cancer. Post EBV infection, the body generates specific serum antibodies like P85-Ab, VCA-IgA, Rta-IgG, and Zta-IgA, acting as valuable adjunct markers for early detection and diagnosis of nasopharyngeal carcinoma [19]. Assessment of EBV-associated serum antibodies is a convenient, non-invasive, speedy, cost-efficient procedure, consistently delivering precise outcomes [19]. This diagnostic method finds utility in routine health screenings among the general populace, high-risk group surveillance, and aids in diagnosing and monitoring therapy for nasopharyngeal carcinoma patients [19].

The primary objective of this research is to evaluate the diagnostic efficacy of EBV-associated serum antibodies—P85-Ab, VCA-IgA, Rta-IgG, and Zta-IgA—both individually and in combination. The results reveal a substantial increase in antibody levels or positivity rates within the NPC cohort compared to the control group, underscoring the robust diagnostic utility of these four markers in nasopharyngeal carcinoma (NPC).

The area under the curve (AUC) for the individual detection of P85-Ab, VCA-IgA, Rta-IgG, and Zta-IgA antibodies are 0.964, 0.916, 0.838, and 0.840, respectively. Among these, the P85-Ab antibody shows the best diagnostic efficacy, outperforming VCA-IgA, Rta-IgG, and Zta-IgA antibodies. In combined detection, the AUCs for the pairs P85-Ab + VCA-IgA, P85-Ab + Rta-IgG, P85-Ab + Zta-IgA, and Rta-IgG + Zta-IgA are 0.990, 0.985, 0.984, and 0.970, respectively. For three-marker combinations, the AUCs of P85-Ab + Rta-IgG + Zta-IgA, P85-Ab + Zta-IgA + VCA-IgA, and P85-Ab + Rta-IgG + VCA-IgA are 0.992, 0.993, and 0.994, respectively. The four-marker combination (P85-Ab + Rta-IgG + Zta-IgA + VCA-IgA) achieved the highest AUC of 0.996. These results indicate that the combined detection of all four antibodies provides optimal diagnostic value.

The significance of this study lies in its contribution to optimizing serological screening strategies for NPC. While classical EBV markers like VCA-IgA and Zta-IgA are widely used, our identification of P85-Ab as a novel marker with superior diagnostic performance provides new insights into biomarker development. The enhanced sensitivity and specificity achieved through combined detection suggest that P85-Ab can serve as a valuable addition to current screening panels. This is particularly important for early detection in endemic and resource-limited areas, where timely diagnosis remains a major challenge. Thus, our findings offer both scientific value and potential clinical application in improving NPC diagnostic strategies.

In the clinical analysis of NPC diagnosis, the sensitivity of individual antibodies P85-Ab, VCA-IgA, Rta-IgG, and Zta-IgA is 93.00%, 75.00%, 59.00%, and 62.00%, respectively, while their specificity is 99.00%, 95.00%, 96.00%, and 88.00%. Among these, the P85-Ab antibody exhibits the highest sensitivity and specificity. By combining P85-Ab with other markers, sensitivity can be further enhanced, effectively improving the diagnostic efficacy for NPC and serving as a serological marker for NPC diagnosis.

Considerable research currently exists on the correlation between EBV antibody levels and the clinical staging of nasopharyngeal carcinoma (NPC), a topic surrounded by significant debate. This study initially investigated the link between the positivity rates of four EBV antibodies and the clinicopathological characteristics in a cohort of 100 NPC patients. The results revealed no substantial association between the positivity rates of serum antibodies P85-Ab, VCA-IgA, Rta-IgG, and Zta-IgA and variables like gender, age, clinical stage, and TNM staging (P > 0.05). Notably, most of the 100 newly diagnosed NPC patients included in this study were in advanced stages (III and IV), largely due to the asymptomatic nature of early-stage NPC and the challenges in its timely detection. This has resulted in a limited representation of early-stage cases, which may introduce bias and affect the generalizability of the findings. Future studies should aim to expand the sample size and improve the inclusion of early-stage patients to enhance the robustness and applicability of the results. In terms of diagnostic performance, although the combination of P85-Ab + VCA-IgA achieved a high AUC (0.990), our results showed that the four-marker combination (P85-Ab + Rta-IgG + Zta-IgA + VCA-IgA) yielded the highest AUC (0.996), along with superior sensitivity and NPV. While its specificity was slightly lower, the overall diagnostic performance was optimal. In regions with high prevalence of nasopharyngeal carcinoma or for screening high-risk populations, maximizing sensitivity and early detection may be prioritized over specificity. Therefore, we believe that the four-marker combination still holds significant clinical value, particularly in applications where diagnostic accuracy is paramount.

Importantly, P85-Ab alone performed significantly better than each of the other single biomarkers (all P < 0.05), confirming its strong diagnostic value as a single marker. When compared with the four-marker panel, the combination showed a P value of 0.088 versus P85-Ab alone, close to marginal significance, suggesting a potential incremental advantage of combining multiple antibodies. This finding highlights the possibility that multi-marker strategies may further enhance diagnostic performance beyond P85-Ab alone. Expanding the sample size and including more early-stage cases will be critical to further validate this trend, representing a promising direction for future research in NPC screening.

In addition to antibody-based markers, EBV-DNA testing is a well-established and highly reliable biomarker that plays a pivotal role in the diagnosis and management of NPC. Considering this, the combined detection of EBV-DNA and P85-Ab represents a promising avenue for further enhancing diagnostic accuracy, and we intend to explore this approach in future studies.

In summary, the P85-Ab antibody demonstrates good sensitivity and specificity when used alone. Combined detection significantly enhances sensitivity while maintaining good specificity and accuracy. The combination of P85-Ab + Rta + Zta + VCA is one of the effective methods for diagnosing NPC. These findings provide important experimental data for the early screening of NPC and hold clinical significance for the diagnosis and treatment of NPC patients.

## Supporting information

S1 File Detection results of indicators in nasopharyngeal carcinoma patients and healthy controls. (XLSX)
